# Death anxiety, moral courage, and resilience in nursing students who care for COVID-19 patients: a cross-sectional study

**DOI:** 10.1186/s12912-022-00931-0

**Published:** 2022-06-13

**Authors:** Fateme Mohammadi, Zahra Masoumi, Khodayar Oshvandi, Salman Khazaei, Mostafa Bijani

**Affiliations:** 1 Chronic Diseases (Home Care) Research Center and Autism Spectrum Disorders Research Center, Department of Nursing, Hamadan University of Medical Sciences, Hamadan, Iran; 2 Department of Nursing, Hamadan University of Medical Sciences, Hamadan, Iran; 3 Mother and Child Care Research Center, Hamadan University of Medical Sciences, Hamadan, Iran; 4 Department of Epidemiology, School of Health , Health Sciences Research Center, Health Sciences & Technology Research Institue, Hamadan University of Medical Sciences, Hamadan, Iran; 5 Department of Medical Surgical Nursing, Fasa University of Medical Sciences, Fasa, Iran

**Keywords:** Anxiety, Death, Courage, Resilience, COVID-19, Nursing students

## Abstract

**Background:**

Being on the frontline of the battle against COVID-19, nurses and nursing students have been under considerable psychological stress and pressure. The present study is done to explore death anxiety, moral courage, and resilience in nursing students caring for COVID-19 patients in the south of Iran.

**Methods:**

The present study is cross-sectional research conducted between September and December 2021. A total of 420 senior nursing students (nursing interns) who were undergoing their clinical training courses were invited to participate in the study by convenience sampling method from three hospitals affiliated with the University of Medical Sciences of Southern Iran. Data were collected using a demographics survey, Nurses’ Moral Courage Questionnaire, Connor-Davidson Resilience Scale, and Templer’s Death Anxiety Scale.

**Results:**

The nursing students participating in this study had a death anxiety mean score of 12.78 ± 1.17**.** The results showed that there was a significant and indirect correlation between death anxiety on the one hand and moral courage (*r* = -0.91, *p* < 0.001) and resilience (*r* = -0.89, *p* < 0.001) on the other in nursing students caring for patients with COVID-19. Also, it was found that there was a significant and direct correlation between the students’ resilience and moral courage scores (*r* = 0.91, *p* < 0.001).

**Conclusion:**

The nursing students caring for COVID-19 patients had experienced high levels of death anxiety in the past few months. Considering the persistence of the COVID-19 crisis in Iran and other countries, there is an urgent need for measures to preserve and improve the physical, mental, and spiritual health of nursing students, enhance their moral courage and resilience and reduce their death anxiety.

## Introduction

The emerging disease of COVID-19 has caused indescribable fear and anxiety across the world in the past three years [1]. The little-known virus started in China and spread to almost all the countries in the world [2, 3]. In this century, the COVID-19 pandemic has proven a major threat to human health. The mysterious nature of COVID-19 and the frequent mutations of the coronavirus have infected more than 250 million people and killed more than 5 million people worldwide to date [4, 5]. The pattern of COVID-19 infections in Iran has been complex. At the pandemic's beginning, after China and Italy, Iran was the third country with the highest infection rate and witnessed several waves that led to high mortality. Up to now, Iran has been hit by five waves of COVID-19, while many countries have seen two [6]. Despite the high public awareness about the infection and its risks, the financial difficulties caused by strict sanctions force the people to go to crowded places for their livelihood, which is the main reason for the repeated emergence of peaks in the number of the infected people [6, 7]. In Iran, over 6,000,000 have been infected with COVID-19, and more than 128,000 have lost their lives to date [7]. The high mortality rate of COVID-19 globally has caused many people to experience great stress, with adverse effects on their social activities and psychological security [8]. Healthcare professionals, the first and most important protectors of public health in the face of this highly infectious disease, have been exposed to various emotional and psychological challenges in this period [9]. The anxiety of death is one of the most important psychological challenges caregivers experience when caring for patients with COVID-19 [10]. Death anxiety is a persistent, abnormal, and morbid fear of death or dying, which is an important concept in nursing [11].

Therefore, due to the importance of this concept in providing health care to patients, death anxiety has been included in the North American Nursing Diagnosis Association (NANDA) criteria as a nursing diagnosis [12].

In recent years, death anxiety in patients has received much attention [13]. Still, the incidence of COVID-19 with high morbidity and mortality has caused caregivers to experience indescribable fear and anxiety of death. The caregivers’ fear and anxiety of death strongly affected their resilience to provide quality care to these patients [11–13]. Resilience is the ability to adapt to life-threatening situations successfully. In other words, resilience is a positive adaptation in reaction to adverse conditions and any attempt to return or achieve a higher level of adaptation [14].

On the other hand, death anxiety can severely affect the moral courage of caregivers. By overcoming their fear and anxiety, they can provide safe and principled care for their patients [15]. Moral courage is defined as the courage to act according to one's ethical values and principles, even at the risk of negative consequences for the individual [16]. Moral courage helps caregivers adhere to the principles and values of professional ethics in caring for the patient with COVID-19.

According to recent studies, healthcare providers who directly contact COVID-19 patients have experienced frustration, stress, burnout, and reduced resilience [17]. Lai et al. (2020) report that healthcare professionals who provide care to COVID -19 patients experience higher levels of anxiety and depression and have problems performing their professional duties and their family responsibilities and daily activities [18]. Likewise, Roy (2020) states that during the COVID-19 crisis, nurses have suffered from stress, anxiety, depression, and post-traumatic stress disorder (PTSD) [19].

On the other hand, Khodaveisi. et al. (2021) report that, even though healthcare professionals have demonstrated signs of high and moderate resilience during the pandemic, they succeeded with courageous efforts to provide quality care to the infected [16].

One of the most prominent care providers in Iran is senior nursing students (nurse interns), who have been caring for COVID-19 patients and other healthcare personnel members. Because of their inexperience, inadequate skills, eagerness to finish their education, uncertainty about the end of the pandemic in Iran, and youth-related emotions, nursing students obviously experience tension and psychological stress than other care providers do. One of the main sources of tension for this population is death anxiety which can adversely affect their performance, resilience, and moral courage. However, no research work has investigated ethical distress, death anxiety, courage, or resilience in nursing students caring for COVID-19 patients. Accordingly, the present study investigates the relationship between death anxieties on the one hand and moral courage and resilience on the other in nursing students who care for COVID-19 patients in Iran. Also, the main research question was” Is there a relationship between death anxiety with moral courage and resilience in nursing students who care for COVID-19 patients? ".

## Methods

### Participants and sampling

The present study is a cross-sectional work of research conducted between September to December 2021 based on the strengthening of the reporting of observational studies in epidemiology statement (STROBE), which is a checklist for observational research.

The sample size for this study was calculated based on the Khodaveisi et al. [16] study with a power of 80% and α = 0.05 by using the formula.

$$n=\frac{{z}_{1-\frac{\alpha }{2}}^{2}{s}^{2}}{{d}^{2}}$$

For sampling, the correspondence author went to the school of nursing affiliated with the university of medical sciences in the west of Iran after receiving ethical approval and got the phone number of senior nursing students in the 7th and 8th semesters of nursing had the inclusion criteria. Therefore, 420 senior nursing students, who were doing internships in three major hospitals affiliated with the school of nursing, central for infectious diseases, heart filer, lung disease, kidney disease, and cancer, were invited and selected through convenience sampling to participate in the study.

The inclusion criteria were senior nursing students in the 7th and 8th semesters of nursing, being willing to participate in the study, being trained in a COVID-specific hospital department, and caring for at least one COVID-19 patient in a clinical environment. The participants who failed to answer more than half of the questions on the questionnaires or did not return the questionnaires were excluded. The participants were asked to complete and submit the demographics survey, Nurses’ Moral Courage Questionnaire, Connor-Davidson Resilience Scale, and Templer’s Death Anxiety Scale on the Internet. A total of 400 participants returned completed questionnaires by email or social networks, with a response rate of 95.23%.

## Measurement

### Demographics survey

The demographics survey addressed the respondents’ age, gender, marital status, financial status, history of caring for COVID-19 patients, and an average number of shifts per month.

### Templer’s death anxiety scale

The Death Anxiety Scale consists of 15 true or false questions, 9 of which earn a score of 1 if the respondent chooses true, and 6 items earn a score of 1 if the respondent chooses false. The total score range is between 1 and 15, with higher scores indicating greater death anxiety. Respondents’ scores are classified as indicating mild (0–6), moderate (7–9), and severe (10–15) anxiety. The scales possess content and construct validity and reliability, with a Cronbach’s alpha of 0.83 [20]. Cronbach’s alpha for this questionnaire in the present study was 0.91.

### Nurses’ moral courage questionnaire

Developed and evaluated by Sadoughi in 2015, the Nurses’ Moral Courage Questionnaire consists of 20 items that address the three dimensions of moral self-realization (9 items), risk-taking (8 items), and ability to defend one’s rights (3 items). The items are scored on a 5-point Likert scale, ranging from always (5) to never (1). For each item, the score is the Likert score multiplied by the value of that item. The minimum and maximum scores are 102 and 510, respectively. A score of 102 to 238 indicates low moral courage, 239 to 374 indicates moderate moral courage, and 375 to 510 indicates high moral courage. The content validity of the questionnaire has been verified by a content validity index (CVI) of 0.87, and its internal consistency has been verified by a Cronbach’s alpha of 0.88. As measured by the test–retest method and calculation of its interclass correlation coefficient, the instrument's reliability has been reported to be 0.87 [21]. Cronbach’s alpha for this questionnaire in the present study was 0.90.

### Connor-Davidson Resilience Scale (CD-RISC)

The resilience scale was developed by Connor and Davidson in 2003. The instrument comprises 25 items that measure five categories: the notion of personal competence (items 10, 11, 12, 16, 17, 23, 24, 25), trust in one's instincts, and tolerance of negative effects (items 6, 7, 14, 15, 18, 19, 20), the positive acceptance of change and secure relationships (items 1, 2, 4, 5, 8), control (items 13, 21, 22), and spiritual influences (items 3 and 9). Scoring is based on a 5-point Likert scale, ranging from “not true at all” = 0 to “true all of the time” = 4. The score range is between 0 and 100 respectively. A score of 0 to 33 indicates low resilience, 34 to 67 indicates average resilience, and above 68 indicates high resilience [22]. The scale's reliability has been verified by a Cronbach’s alpha of 0.91 in Iran [23]. Cronbach’s alpha for this questionnaire in the present study was 0.93.

### Ethical considerations

The ethics committee has approved the present study of the ethical code (IR.UMSHA.REC.1401.223). The present study was conducted in terms of the revised Declaration of Helsinki principles, a statement of ethical principles that directs physicians and other participants in medical research involving human subjects. All participants were informed of the study's objectives and asked to sign the consent form. They were also assured that all their information would remain anonymous and confidential.

### Statistical methods

After being collected, the data were analyzed in SPSS v. 22 using descriptive statistics (frequency, percentage, mean, and standard deviation). To investigate the relationship between the participants’ death anxiety and their resilience, moral courage, and demographic variables, the researchers used the chi-square test, t-test, and ANOVA. The level of significance was set at *P* < 0.05. The demographic variables, resilience, and moral courage that correlate with death anxiety (*p* < 0.25) were entered into a multiple linear regression model with a backward design. Before using multiple linear regression, the researchers tested the following assumptions: normality of the data, homogeneity of variance, and independence of residuals.

## Results

Participants in this study were aged 19 to 35 years, years with a mean age of 25.17 ± 2.41. Most of the nursing students were male, 220 (55%), and had six months of experience with an average of 21 shifts per month. The findings also showed a statistically significant relationship between death anxiety and work experience, age, and financial status (Table 1).

**Table 1:** The participants’ demographic characteristics and death anxieties score

Demographic variables		N (%)	Death anxieties SD ± Mean	*P* -Value
***Age (year)***	19–24	157(39.25)	13.94 ± 1.13	**0.021**
	25–30	124(31.00)	11.43 ± 1.21	
	31–35	119(29.75	10.57 ± 1.54	
***Gender***	Female	180(45.00)	13.58 ± 1.39	0.471
	Male	220(55.00)	10.31 ± 1.14	
***Marital Status***	Single	270(67.50)	12.05 ± 1.62	0.493
	Married	130(32.50)	12.41 ± 1.24	
***financial status***	< 100 $	250(62.50)	10.13 ± 1.57	**0.018**
	100–200 $	58(21.25)	12.78 ± 1.34	
	> 200 $	65(16.25)	13.98 ± 1.68	
***Work experience***	6–12 months	167(41.75)	13.58 ± 1.14	**0.017**
	13–18- months	172(43.00)	11.64 ± 1.39	
	19–24 months	61(15.25)	10.08 ± 1.52	

### Death anxiety, moral courage, and resilience in the nursing students

In this study, the nursing students reported a death anxiety score of 12.78 ± 1.17, a moral courage score of 240.76 ± 1.62, and a resilience score of 74.54 ± 2.17 in caring for patients with COVID-19 (Table 2).

**Table 2:** The means and standard deviations of the participants’ death anxieties, moral courage and resilience in nursing students

Variable	dimensions	Mean ± SD (Each dimension)	Mean ± SD (Total)
**Moral courage**	Moral self-actualization	230.15 ± 1.78	240.76 ± 1.62
	Risk taking	251.83 ± 1.24	
	Ability to defend the right	240.32 ± 1.85	
**Resilience**	Perception of individual competence	71.65 ± 2.12	74.54 ± 2.17
	Spiritual effects	75.47 ± 1.98	
	Adaptability to change	75.98 ± 2.54	
	Tolerance of negative affect	76.43 ± 2.13	
	Recognition of limits to control	73.17 ± 2.11	
**Death anxieties**	Without dimension	12.78 ± 1.17	12.78 ± 1.17

### Relationship between death anxiety, moral courage, and resilience in the nursing students

The findings in the present study revealed that there is a strong and direct correlation between death anxiety with moral courage (*r* = -0.91, *p* < 0.001) and there is a strong and indirect correlation between death anxiety with resilience (*r* = -0.89, *p* < 0.001) in nursing students caring of patients with COVID-19. Also, the results showed a strong and direct correlation between the score of resilience and moral courage (*r* = 0.91, *p* < 0.001) (Table 3).

**Table 3:** Relationship between death anxieties, moral courage and resilience in nursing students

*Death anxieties*	*Moral* courage	*r* = -0.91	*p* < 0.001
*Death anxieties*	*Resilience*	*r* = -0.89	*p* < 0.001
*Resilience*	*Moral* courage	*r* = 0.91	*p* < 0.001

### Predictors of death anxiety in nursing students who cared for COVID-19 patients

The variables of moral courage, resilience, work experience, age, and financial status which with a *p*-value of smaller than 0.25, were entered into multiple linear regressions with the backward technique. These variables remained in the model and accounted for about 68.12% of the death anxiety variance in the nursing students who cared for COVID-19 patients (Table 4).

**Table 4:** The predictor variables of death anxieties in nursing students who caring for patients with COVID-19

*Factors*	*Non-standard coefficients*		*standard coefficients*	T	*P* -Value
	***B***	**Std. Error**	***β***		
***Moral courage***	2.88	1.78	0.80	1.61	0.001
***Resilience***	2.65	1.64	0.74	1.61	0.001
***Work experience***	1.34	1.01	0.51	1.33	0.032
***financial status***	1.32	1.03	0.39	1.28	0.036
***Age***	1.18	1.07	0.31	1.10	0.041

Adjusted R2: 68.12%

## Discussion

The nursing students who cared for COVID-19 patients had a high death anxiety, moral courage, and resilience. There was a significant and indirect correlation between death anxiety on the one hand and moral courage and resilience on the other and a significant and direct correlation between resilience and moral courage. In addition, the variables of moral courage, resilience, work experience, age, and financial status accounted for about 68.12% of the death anxiety variance in the nursing students who provided care to COVID-19 patients.

Although several studies have investigated work stress, knowledge, and awareness in nurses caring for patients with COVID-19, no article was found to have investigated death anxiety, moral courage, or resilience in nursing students who care for COVID-19 patients. As a result, there was not much research that researchers could compare the present study's findings with; thus, the researchers used other studies that measured the levels of death anxiety, moral courage, and resilience in nurses caring for patients with COVID-19 or patients with special diseases.

The death anxiety score of the nursing students in the present study was reported high, which is considered to be high. Similarly, the results of a study by Maqbalia et al. (2021) in Iran showed that the nurses who were in practice in hospital units where COVID-19 patients were hospitalized had high levels of death anxiety. There was also a significant correlation between the nurses’ death anxiety and their psychological tensions and resilience [24]. Iran is among the top 10 countries most affected by COVID-19. Nurses in practice in COVID-19 wards in Iran have already seen five major waves of infection. The complicated and stressful conditions involved in caring for COVID-19 patients can increase death anxiety in nursing students who have never experienced a crisis of these proportions and do not have enough professional experience, skills, and psychological capacity to cope with it.

According to a study by Mohammadi et al. (2020), nurses experienced immense psychological stress and anxiety in COVID -19 due to an increase in the number of infected death of their young colleagues. The latter had no history of an underlying disease and dying.

Consistent with the present study, these nurses have reported moderate anxiety and moderate resilience, and there was an indirect relationship between resilience and anxiety [25]. Similarly, Ozguc et al. (2021) reported that nurses in COVID-19 wards suffered from high levels of death anxiety. The researchers also found a significant correlation between death anxiety on the one hand and age, gender, work experience, and resilience skills on the other, which is consistent with the findings of the present study [26]. On a similar note, the results of a study by Dadgari et al. (2015) demonstrated that ICU nurses who cared for COVID-19 patients experienced high levels of death anxiety. The results also showed a significant correlation between the nurses’ death anxiety on the one hand and gender, work experience, and degree of resilience on the other. [27].

According to Savitsky et al. (2020), because of their inadequate resilience skills and fear of contracting COVID-19 and losing their lives, nursing students have experienced severe anxiety and other emotional and psychological crises [28]. Similarly, Baluwa et al. (2021) and also, Kim et al. (2021) reported that nursing students caring for COVID-19 patients experienced high levels of workplace anxiety that adversely affected their clinical performance and resilience [29, 30]. The studies of Yiğit & Açıkgöz (2021) and Karabağ Aydın & Fidan (2021) also demonstrated that, during the COVID-19 pandemic, nurses had been exposed to significant levels of death anxiety. There was an indirect relationship between resilience and anxiety, which agrees with the present study's findings [31, 32]. Likewise, Zukhra et al. (2021) found that nursing students have experienced high anxiety and psychological stress levels during the current pandemic. Accordingly, healthcare policymakers are recommended to provide nurses and nursing students with more psychological support to improve their psychological skills, mental health, and spiritual health [33]. Cheng et al. (2021) showed that improving nursing students’ spiritual health and psychological capacities, including resilience, reduce their death anxiety during the COVID-19 pandemic [34].

In the present study, even though they had been exposed to difficult conditions, the nursing students who provided care to COVID-19 patients achieved above-average moral courage mean score. The crisis had not caused them to ignore their clinical duties. They sacrificed their safety to provide care to patients. They remained committed to their ethical and professional responsibilities to save the lives of the infected people in the critical circumstances caused by COVID-19. Khodaveisi et al. (2021) found the moral courage mean score of nurses who were in practice in COVID-19 wards to be high [16]. They also found a significant correlation between the nurses’ moral courage and resilience, which is consistent with the present study's findings. On a similar note, Gibson et al. (2020) found a direct correlation between nursing students’ resilience and moral courage [35]. Cerit et al. (2021) reported a significant negative correlation between nurses’ death anxiety and ethical sensitivity: elevated ethical sensitivity correlated with reduced death anxiety in the nurses [36].

The present study results showed the students’ resilience mean score to be average. Similarly, Labrague (2021) study found the resilience of nursing students during the COVID-19 pandemic to be average. Since resilience acts as a major mediator in individuals' psychological well-being and life satisfaction, special attention should be given to the psychological capacities and resilience skills of nursing students in the face of workplace stressors. Training this population in coping strategies is one way to enhance their resilience. The studies mentioned above also reported a significant correlation between the subjects’ resilience to student anxiety and especially death anxiety, which agrees with the present study's findings [37].

## Limitations

The present study evaluated the nursing students in the south of Iran. It is, therefore, recommended that future research address different societies and use larger samples to achieve a more accurate measure of nursing students’ death anxiety, moral courage, and resilience during the COVID-19 crisis so that healthcare policymakers and managers can develop more comprehensive plans to cope with the current and similar crises.

## Strengths

The present study was the first attempt at investigating death anxiety, moral courage, and resilience in nursing students caring for COVID-19 patients in Iran. Other strengths of the study are its relatively large sample size and use of specific questionnaires.

## Conclusion

In the present study, nursing students who care for COVID-19 patients have been exposed to high degrees of death anxiety in recent months. Yet, despite the considerable tension and anxiety involved in caring for this group of patients for a relatively long time, nursing students have remained committed to performing their clinical duties and displayed great moral courage and resilience. According to the persistence of the COVID-19 crisis in Iran and other countries in the coming months, measures must be taken to preserve and improve the physical, mental, and spiritual health of nursing students, enhance their moral courage and resilience and relieve their death anxiety.

## Data Availability

The datasets generated and/or analysed during the current study are not publicly available due to the necessity to ensure participant confidentiality policies and laws of the country but are available from the corresponding author on reasonable request.

## Abbreviation

NANDA: North American Nursing Diagnosis Association
